# Molecular Characterization of Bacterial Agents Causing External Ocular Infections Isolates of Patients in a Third Level Hospital

**DOI:** 10.3390/pathogens12111294

**Published:** 2023-10-29

**Authors:** Emilio Mariano Durán-Manuel, Juan Manuel Bello-López, Aranza Denisse Salinas-Bobadilla, Cruz Vargas-De-León, Nayeli Goreti Nieto-Velázquez, Mario Adán Moreno-Eutimio, Rodolfo Pastelin-Palacios, Claudia Camelia Calzada-Mendoza, Dulce Milagros Razo Blanco-Hernández

**Affiliations:** 1 Hospital Juárez de México, Mexico City 07760, Mexico; 2 Sección de Estudios de Posgrado e Investigación, Escuela Superior de Medicina, Instituto Politécnico Nacional, Mexico City 11340, Mexico; 3 Facultad de Estudios Superiores Zaragoza, Universidad Nacional Autónoma de México, Mexico City 09230, Mexico; 4 Facultad de Química, Universidad Nacional Autónoma de México, Ciudad Universitaria, Mexico City 04510, Mexico

**Keywords:** ocular infections, antimicrobial resistance, phenotype, genotype, bacteria, *Staphylococcus*

## Abstract

Empirical use of antibiotics in the treatment of eye infections leads to bacterial pathogens becoming resistant to antibiotics; consequently, treatment failure and eye health complications occur. The aim of this study was to describe the phenotype and genotype of the resistance and adherence of bacterial agents causing eye infections in patients at Hospital Juárez de México. An observational, prospective, cross-sectional, and descriptive study was carried out in patients with signs and symptoms of ocular infection. Bacterial agents were isolated and identified by classical microbiology and mass spectrometry. Antibiotic resistance and adherence profiles were determined. Finally, resistance (*mecA*/*SCCmec*) and virulence (*icaA* and *icaD*) genes were detected in the Gram-positive population. The results showed that blepharitis was the most prevalent condition in the study population. A MALDI-TOF analysis revealed that *Staphylococcus* and *Pseudomonas* genus were the most prevalent as causal agents of infection. Resistances to β-lactams were detected of 44 to 100%, followed by clindamycins, aminoglycosides, folate inhibitors, and nitrofurans. A multiple correspondence analysis showed a relationship between *mecA* genotype and β-lactams resistance. The identification of *SCCmecIII* and *SCCmecIV* elements suggested community and hospital sources of infection. Finally, the coexistence of *icaA^+^*/*icaD^+^*/*mecA*(*SCCmecIII*) and *icaA^+^*/*icaD^+^*/*mecA*(*SCCmecIV*) genotypes was detected in *S*. *aureus*. The identification of resistant and virulent isolates highlights the importance of developing protocols that address the timely diagnosis of ocular infections. Herein, implications for the failure of antimicrobial therapy in the treatment of ocular infections in susceptible patients are analysed and discussed.

## 1. Introduction

Most superficial ocular infections have a self-limited course due to the mechanisms of the ocular immune response; however, failure of this response can lead to bacterial colonisation of ocular tissues [1,2]. It has been shown that ocular tissues, being rich in biomolecules—mainly proteins—allow pathogen proliferation to occur easily even though the eye is an immune-privileged organ [3]. In bacteriological terms, ocular infections can be mono- or polymicrobial and are associated with factors such as contact lens wear, trauma, surgery, systemic disease, advanced age, dry eye, chronic nasolacrimal duct obstruction, and previous ocular infections [3,4]. Bacterial infection is the most common complication, where keratitis, the most serious ocular infection, is the leading cause of corneal blindness and the second most common cause of blindness worldwide [5,6,7]. Therefore, antimicrobial therapy for these types of infections should be targeted through the selection of resistant bacterial pathogens to use the correct antimicrobial during therapy [8]. However, the empirical use of topical antibiotics to treat these types of infections increasingly leads to bacterial pathogens that are isolated, becoming resistant to various antibiotics with the consequent treatment failure and complications that this could have on visual health [9].

Detecting the specific aetiological agent, as well as understanding the ocular microbiota and its relationship with the host and the patient’s comorbidities, allows for optimal management of these infections [10,11,12]. Gram-positive bacteria are the main contributors to bacterial eye infections, such as *Staphylococcus aureus* and coagulase-negative *Staphylococcus*, where some species belonging to the skin microbiota are found, e.g., *S. epidermidis* and *S. saprophyticus* [13,14]. Other bacterial genera that have been reported in smaller proportions are *Streptococcus* (*S. pneumoniae*, *S. pyogenes*, and *S. viridians*) [15]. Finally, other positive bacteria such as *Corynebacterium diphtheriae*, *Bacillus cereus*, *B. thuringiensis*, *B. subtilis*, *B. mycoides*, *B. pumilis*, *B. flexus*, and *Clostridium* have been recognised as causative agents of eye infections [16,17,18].

On the other hand, clinically important Gram-negative bacteria have been isolated as causative agents of infections in patients with comorbidities. This group of pathogens are constituted by lactose-fermenting and non-fermenting bacteria, such as *K. pneumoniae*, *Proteus*, *Citrobacter*, and *P. aeruginosa* [19]. In relation to antimicrobial resistance, bacteria have been identified as resistant to multiple drugs that are used in the therapy of eye infections, such as those belonging to the β-lactam families, aminoglycosides, phenicols, quinolones, tetracyclines, macrolides, and lipopeptides [9,20]. This shows the risk associated with failure of antimicrobial therapy and, consequently, fatal conditions due to excessive pathogen proliferation, which can lead to visual organ loss. In contrast, knowing that Gram-positive bacteria are reported as the main causative agents of ocular infections, the detection of methicillin-resistant *S. aureus* (MRSA) strains is of utmost relevance, as they are epidemiologically relevant due to their high resistance to methicillin and other β-lactam antibiotics [21,22]. 

This highlights the importance of having laboratory tools to address ocular infectious problems that are still a challenge for many developing countries. The aim of this work was to determine the bacteriological diversity of the causative agents of eye infections isolated from patients at Hospital Juárez de México, to know their drug resistance profile and the genetic basis of their β-lactam resistance (*mecA* gene and *SCCmec* element) and virulence (*icaA* and *icaD* genes). Implications for failures in antimicrobial therapy in the treatment of ocular infections by antibiotic-resistant and virulent bacteria in susceptible patients are analysed and discussed.

## 2. Materials and Methods

### 2.1. Ethical Considerations

The Institutional Committee of Research, Ethics, and Biosafety from HJM approved the protocol under the registration number HJM 023/21-I in accordance with the Regulation of the General Health Law on Research for Health. Informed consent was obtained from the participants prior to their recruitment into the study.

### 2.2. Study Population with External Ocular Infection

A population consisting of 110 patients from HJM with external ocular infection was included in the study during the period between September (2021) and January (2022). These patients met the inclusion criteria for external ocular infection (foreign body sensation, itch, erythema, blurred vision, secretions, tearing, eye volume increased). According to external ocular infection, the patient population was classified by blepharitis, conjunctivitis, dacryocystitis, or keratitis. For microbiological diagnosis, in aseptic conditions, patients were sampled through the use of Amies transport medium without charcoal of exclusively corneal scrapings of the site infection, and the samples were transported at 4 °C to the research laboratory to determine their microbiological culture. Additionally, demographic data were obtained from medical records to describe the patient population.

### 2.3. Isolation of Bacteria from External Ocular Infection Cases

Clinical samples were cultured on selective media MacConkey, Mannitol Salt, and Sabouraud dextrose agar; moreover, rich media such as sheep blood and brain heart infusion agar were used. The culture media were incubated aerobically and anaerobically at 37 °C for 24–48 h. Additionally, incubation for one more week was carried out to allow the growth of slow growing/fastidious organisms. Bacterial morphotypes were identified to detect mono and polymicrobial infection and were purified in LB-agar and growth in LB-broth, then frozen in glycerol (50%) and stored at −70 °C for future experiments. 

### 2.4. Bacterial Identification by Mass Spectrometry MALDI-TOF 

Bacterial isolates were identified by the direct analysis of whole bacterial cells using matrix-assisted laser desorption/ionization-time of flight mass spectrometry (MALDI-TOF MS) by the Facultad de Química of Universidad Nacional Autónoma de México (UNAM). For this purpose, all strains were streaked in LB agar and incubated overnight at 37 °C, and single colonies were subjected to identification by using a Bruker MALDI Biotyper (Bruker Daltonik, Germany) according to the manufacturer’s instructions. The criteria to best match with the identification protocol was bacterial strains with score values above 2.0 (down to 3) for high-confidence identification.

### 2.5. Susceptibility/Resistance Assays

Antimicrobial susceptibility testing was performed by using the disk diffusion method on Mueller–Hinton agar plates according to the guidelines set by “The Clinical and Laboratory Standards Institute” (CLSI, 2022) [23]. The antimicrobial resistance was carried out for eighteen antimicrobials grouped in twelve families for Gram-negative and Gram-positive bacteria (antibiotics recommended by CLSI). *Pseudomonas aeruginosa* ATCC 27853, *E. coli* ATCC 25922, and *S. aureus* ATCC 25923 were used as controls. The results were inferred as susceptible or resistant by measuring the diameter of the inhibition zone according to the criteria specified by the CLSI (2022). The frequency of antibiotic sensitive, intermediate, and resistance were calculated and represented in percentages.

### 2.6. mecA Gene and Mobile Genetic Element SCCmec Characterization

To identify the genetic background associated with methicillin-resistant strains, the *mecA* gene and the internal control (*16S rRNA* gene) were amplified by duplex-PCR according to Acosta-Peréz et al., 2012, by using mecA-plus/mecA-minus and 16S-plus/16S-minus primers, respectively [24]. The size of the PCR products is shown in Table 1. The inclusion of an internal control (16S rRNA gene) was justified by the fact that the search for genes by endpoint PCR is conditioned to possible failures in the assays, such as contamination of PCR mixture and others. This guarantees the absence of interest genes in the study strains. Isolates positive to the *mecA* gene were subjected to a second amplification assay by multiplex-PCR using a set of four pairs of primers to identify the mobile genetic element *SCCmec* (type I, II, III, IV, and V), according to Boye et al. (2007) [25] (Table 1). The multiple identification of the PCR products of the molecular targets indicated in Table 1 (*ccrA2-B*, *ccrC*, *IS1272*, and *mecA-IS431*) allowed the assignment of the various five types of *SCCmec* in the isolates studied. *Staphylococcus aureus* ATCC 43300 was used as a positive control for *mecA* and *SCCmec* element (type II).

### 2.7. icaA and icaD Gene Detection in Staphylococcus Strains

The genotypes involved in biofilm formation (exopolysaccharide poly-N acetylglucosamine and GlcNac-transferase) in *Staphylococcus* strains isolated from ocular infection cases were studied through end-point PCR amplification for the detection of the *icaA* and *icaD* genes, according to Rohde et al. (2001), by using icaA-F/icaA-R and icaD-F/icaD-R primers [26] (Table 1). *Staphylococcus aureus* ATCC 43300 was used as a positive control for *icaA* and *icaD* genes carried in full operon *icaADBC*.

### 2.8. In Vitro Biofilms Formation in icaA^+^/icaD^+^ Strains

The biofilm-forming ability was performed in *icaA^+^*/*icaD^+^* strains according to previous reports with minor modifications [27,28]. Briefly, strains carrying *icaA* and *icaD* genes (*n* = 10) were cultured in 3 mL of LB broth with shaking at 37 °C. Overnight cultures were adjusted to 0.5 McFarland nephelometer in LB broth plus 0.2% glucose, then inoculated into wells of a sterile 96-well polystyrene microtiter flat bottom plate (per triplicate) (Thermo Electron Corporation, Corning, NY, USA). The plates were sealed and aerobically incubated at 37 °C for 48 h. Later, the planktonic forms were removed and the plates were washed three times. The remaining biofilms were quantified by measuring the OD_600_ of the supernatant by using an Epoch, BioTek spectrophotometer (Vermont) following biofilm solubilization with 200 mL of 30% CH_3_COOH. Uninoculated well and *S. aureus* (*icaA*^−^/*icaD*^−^) genes were used as negative controls, and *S. aureus* 43300 (*icaA^+^*/*icaD*^+^) was used as a positive control.

### 2.9. Statistical Analysis

A multiple correspondence analysis (MCA) was performed to better visualize the association between the presence of the *mecA* gene in the Gram-positive bacteria population and their antimicrobial resistance to β-lactam antibiotics: AM (ampicillin), PE (penicillin), CF (cephalothin), CFX (cefotaxime), CTX (cefuroxime), FOX (cefoxitin), and CB (carbenicillin). Additionally, MCA was performed to determine possible clusters and associate them with the *mecA* gene presence. The SPSS v.21.0 software and the R Statistical Software v.4.1.3. were used.

## 3. Results

### 3.1. Study Population with Ophthalmic Infection

During the period between September (2021) and January (2022), 122 patients with signs and symptoms of external ocular infection attended the Ophthalmology department of Hospital Juárez de México (HJM) for diagnosis and treatment. Only 110 patients were included in this study. A demographic analysis of the included population showed that the mean age was 58.28 ± 18.74 with a minimum of 2 months and a maximum of 99 years of age. The population distribution by sex showed that 59.09% (*n* = 65) and 40.90% (*n* = 45) were female and male, respectively. Diagnosis derived from clinical inspection showed that blepharitis was the most common presentation in the population (*n* = 102/92.7%), followed by conjunctivitis (*n* = 6/5.45%), dacryocystitis (*n* = 1/0.9%), and keratitis (*n* = 1/0.9%). The most frequent symptomatology was represented by an itchy, foreign body feeling; burning; and secretion. 

Table 2 showed the description of the signs/symptoms in patients with external ocular infections. In relation to the analysis of comorbidities, type 2 *diabetes mellitus* and systemic blood pressure were the most prevalent, followed by a history of ocular surgery and cancer. Table 3 shows the description of the comorbidities in patients with external ocular infections from HJM. Empirical treatment with erythromycin was given to 42 subjects (38.2%), tobramycin to 30 cases (27.2%), ocular lavage to 18 (16.4%), and the rest with different quinolones and some other antibiotics. Of these treatments, the susceptibility analysis showed that only 30% of the treatment was adequate, as the isolated pathogen was sensitive to the treatment given empirically; while 62.7% of the subjects were given an antibiotic to which the isolated pathogen was resistant, and 4.5% did not require antibiotic treatment.

### 3.2. Bacterial Identification by Mass Spectrometry MALDI-TOF

**Figure 1:**
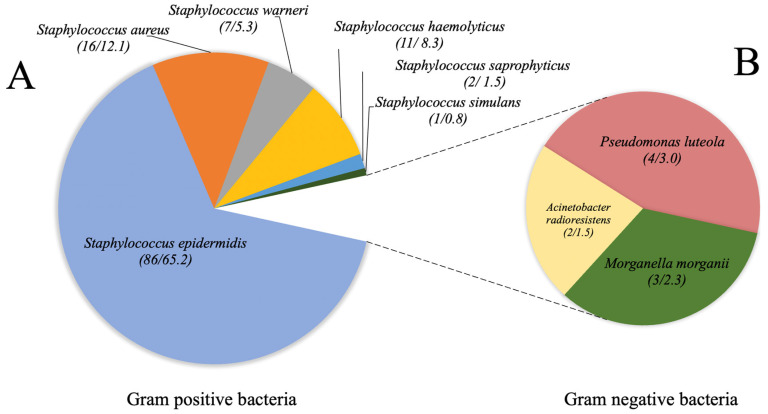
Taxonomic distribution of bacterial strains by genus and species identified by mass spectrometry MALDI-TOF from external ocular infection cases from Hospital Juárez de México during the period between September (2021) and January (2022). (**A**) Gram positive bacteria population. (**B**) Gram negative bacteria population.

The bacteriological analysis revealed that only 93.6% (*n* = 103) of the clinical samples showed bacterial growth with clinical value. The absence of bacterial growth on bacterial cultures in the remaining 6.4% patients (*n* = 7) was confirmed by repeat cultures. Polymicrobial and monomicrobial cultures were identified in 14.6% (*n* = 15) and 85.4% (*n* = 88), respectively. Yeast and filamentous fungi were not isolated. A total of 132 strains were classified as Gram-positive and Gram-negative bacteria. The MALDI-TOF analysis showed that *Staphylococcus* was the most prevalent genus, where *S. epidermidis* was the predominant species. In contrast, for the Gram-negative group, *Pseudomonas* genus was the most common causative agent of infection. Figure 1 shows the distribution of isolates by genus and species identified by MALDI-TOF technology in cases of external ocular infection.

### 3.3. Susceptibility/Resistance Assays

**Figure 2:**
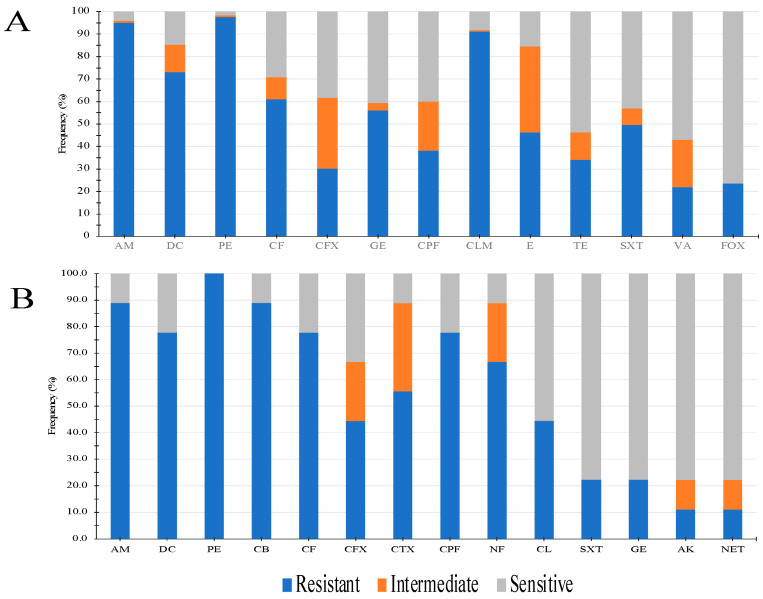
Antimicrobial resistance of strains isolated from external ocular infection cases. (**A**). Total Gram-positive bacteria (**B**). Total Gram-negative bacteria. Antimicrobial families tested: AM (ampicillin), DC (doxycycline), PE (penicillin), CF (cephalothin), CFX (cefotaxime), CTX (cefuroxime), GE (gentamicin), CPF (ciprofloxacin), CLM (clindamycin), E (erythromycin), TE (tetracycline), SXT (trimethoprim/sulfamethoxazole), VA (vancomycin), FOX (cefoxitin), CB (carbenicillin), NF (nitrofurantoin), CL (chloramphenicol), AK (amikacin), NET (netilmicin).

For this analysis, all isolates were divided into two groups (Gram-positive and Gram-negative) and tested for antimicrobial resistance according to the standards established by CLSI (2022). In general, Gram-positive isolates were predominantly resistant to b- lactam antibiotics, followed by clindamycins, aminoglycosides, and folate metabolism inhibitors. Conversely, antibiotics from the tetracycline, nitrofuran, and glycopeptide families showed higher antimicrobial effectiveness. Interestingly, a part of this bacterial population showed resistance to cefoxitin (*n* = 29), a phenotype of epidemiological surveillance due to its association with methicillin resistance (Figure 2A). For isolates from the second group (Gram-negative), β-lactam antibiotics showed less activity, followed by nitrofurans. Finally, antibiotics from the aminoglycoside, folate metabolism inhibitor, and phenolic families showed the highest antimicrobial activity (Figure 2B). The antimicrobial resistance phenotypes of the two bacterial populations isolated from external eye infection cases are shown in Figure 2.

### 3.4. mecA Gene and Mobile Genetic Element SCCmec Characterization

The genetic background associated with methicillin resistance (*mecA* and *SCCmec* element) was analysed by detecting the gene encoding the penicillin-binding protein PBP2a (*mecA*) and the *Staphylococcal Chromosomal Cassette mec* (*SCCmec*). The results revealed that isolates showing resistance to this antibiotic (*n* = 29) were able to detect the *mecA*/*SCCmec* genotype. Genetically different isolates showed the presence of this gene; however, in *S. epidermidis* strains it was more prevalent. Regarding the *SCCmec* genetic elements, type III was the most prevalent in *S. epidermidis*, followed by type IV. Table 4 shows the distribution of the *mecA* gene and the *SCCmec* genetic element by genus and species identified in positive bacteria isolated from cases of external ophthalmic infections.

### 3.5. icaA and icaD Gene Detection and Biofilm Formation in Staphylococcus

The genotype related for bacterial adherence was detected through amplification of the *icaA* and *icaD* genes in *Staphylococcus* isolates. The results showed a prevalence of 8.9% (*n* = 11) for both genes analysed. The *icaA^+^*/*icaD^+^* genotype was predominantly identified in *S. aureus* (*n* = 9), followed by *S. epidermidis* (*n* = 2). Interestingly, the coexistence of the genotype *icaA^+^*/*icaD^+^*/*mecA*(*SCCmecIII*) and *icaA^+^*/*icaD^+^*/*mecA*(*SCCmecIV*) was identified in three *S. aureus* isolates. Finally, the results on biofilm formation ability showed that 100% were classified as strong biofilm producers.

### 3.6. Multiple Correspondence Analysis for mecA Gene and Antimicrobial Resistance

**Figure 3:**
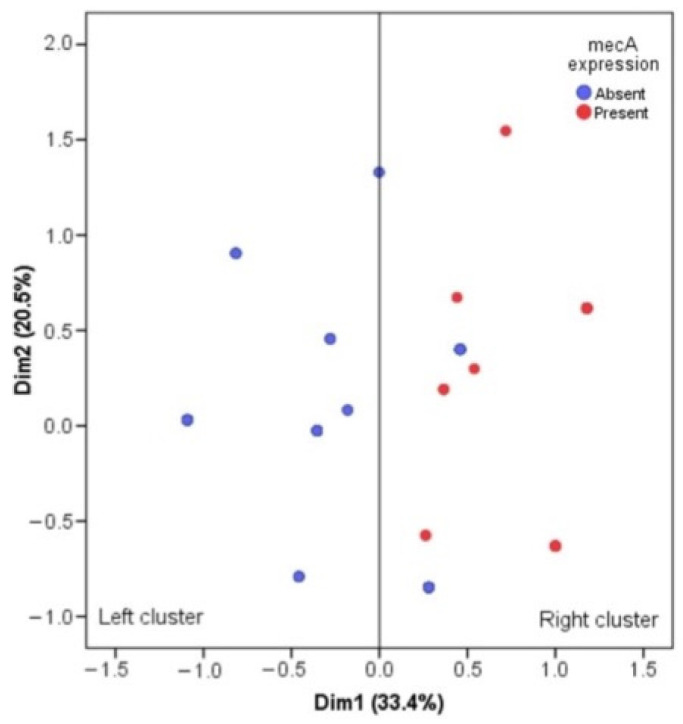
Multiple correspondence analysis for *mecA* gene and antimicrobial resistance to β-lactamics in *Staphylococcus* strains isolated from external ocular infection cases from Hospital Juarez de Mexico.

We implemented a multiple correspondence analysis (MCA) to perform a dimensional reduction of seven antibiotics belonging to the b-lactam family (AM, PE, CF, CFX, CTX, FOX, and CB) in the Gram-positive population. Figure 3 shows that the first two dimensions explain 53.9% of the total inertia; this shows that two clusters are formed, which we call the left cluster and the right cluster. Particularly, these clusters are associated with only the presence of the *mecA* gene (*p*-value < 0.001); 29 (100%) of the 29 Gram-positive bacteria that carried the *mecA* gene are found in the right cluster and 57 (60.6%) of 94 strains that did not carry the *mecA* gene are found in the left cluster. Therefore, it is concluded that there is a correspondence between the presence of the *mecA* gene and the antibiotic resistance of the b-lactam family (Figure 3).

## 4. Discussion

According to international reports, eye infections represent one of the main ophthalmological problems with high morbidity and, in critical situations, these can compromise vision and lead to blindness [19,29,30]. Although mortality associated with these infectious diseases is low, the identification of causative agents, susceptibility profiles, and antimicrobial resistance should be a necessary activity. This is to provide patients with specific antimicrobial treatments and thus avoid the chronicity of infectious events and the emergence of strains resistant to multiple antibiotics. Therefore, in the present study we investigated the bacterial diversity of the causative agents isolated from ocular infections, as well as the phenotypic and genetic basis of resistance and virulence in a cohort of patients treated at the Ophthalmology department of HJM. As shown, blepharitis was the main condition detected in the study population, being recognised as the most common regardless of age, ethnicity, and sex [31]. Although this condition is not vision-threatening, if left untreated it can cause keratopathy, neovascularisation, corneal ulceration, and others [32,33,34,35]. Comorbidities such as *diabetes mellitus*, high blood pressure, as well as chronic degenerative diseases such as cancer are added as susceptibility factors for the acquisition of eye infections. In a previous study, Brown et al. (2002) identified a 64.1% rate of comorbidities in patients with eye disease, where *diabetes mellitus*, heart disease, cancer, stroke and/or renal failure were identified [36]. Coincidentally with our work, *diabetes mellitus* together with arterial hypertension were the most prevalent comorbidities in the population analysed. Regarding bacteriological findings, Gram-positive b-lactam-resistant isolates (AM, PE, CF, CFX, CTX, FOX, and CB) were predominant, where *S. epidermidis* was the species most frequently associated with the infectious events studied. In this context, it has been reported that *S*. *epidermidis* can cause infections such as conjunctivitis, blepharitis, corneal ulcers, and endophthalmitis, in addition to being one of the species frequently isolated from external ocular infections [36,37,38,39]. It is important to consider that every healthy person has ocular surface microbiota, which can vary according to age, environment, and geographic location, among others [2,40,41].

As part of the ocular microbiota, the genus *Staphylococcus* and the species *S. aureus* and *S. epidermidis* are recognised as the most predominant. Nevertheless, these same microorganisms can cause infection of the ocular tissue due to imbalances in the immune and defence mechanisms of the eye itself, for example, physical barriers (eyelids and eyelashes), tear film with lactoferrin, lysozyme, and IgA (antimicrobial and anti-inflammatory) [2,42]. Such is the impact of this imbalance in the microbiota–host relationship that up to 73% of the total bacterial pathogens identified have been reported as part of the microbiota [43]. In contrast to our work, some studies indicate that the most prevalent microorganism in these infections is *S. aureus*, where a high percentage are methicillin-resistant *S. aureus* (MRSA) [44,45]. As shown in Table 4, the species that showed the greatest resistance to methicillin in this study was *S. epidermidis*, and the genetic background associated with resistance to this antibiotic (*mecA*) was also demonstrated. In addition, the multiple correspondence analysis for the *mecA* gene and antimicrobial resistance confirmed the close relationship between the presence of this genetic marker and antimicrobial resistance to antibiotics of the b-lactam family (Figure 3). In this context, to provide more information on the origin of the infectious processes (community or hospital), all cefoxitin-resistant isolates were tested for the *SCCmec* genetic element. As shown in Table 4, the *SCCmec* type III genetic element was the most prevalent, followed by type IV, which was also heterogeneously distributed in all species. However, as *S. epidermidis* was the most abundant species, it showed the highest frequency of the *SCCmec* element. With the information presented above, we can conclude that the infectious origins of the cases studied are of hospital and community type, since the literature has shown that *SCCmec* element types I, II, and III are associated with hospital origins.

In addition, isolates carrying the *SCCmec* type II element have been shown to have other properties, such as low toxin production [46]. In contrast to isolates carrying type IV-V *SCCmec*, genetic elements are considered to be community-acquired pathogens that can produce high levels of toxins [47]. Future work will be aimed at elucidating the presence of genetic markers involved in virulence in this type of isolate according to the methodology described by Panda et al. (2014) [48]. Recent studies have shown the relevance of the coexistence of resistance and virulence factors in *Staphylococcus* isolates, with the *icaADBC* operon and the *mecA* gene being the most studied [49,50]. The identification of genotypes *(icaA^+^*/*icaD^+^*/*mecA*(*SCCmecIII*) and *icaA^+^*/*icaD^+^*/*mecA*(*SCCmecIV*) in *S. aureus* isolates provides information on the possible persistence of microorganisms in the host by high adherent property and b-lactam resistance, as it has been suggested that the exopolysaccharides synthesised by the *icaADBC* operon provide physical protection to the entry of antibiotics into these biological structures [51,52]. 

Conversely, the other Gram-positive species identified in the present work, albeit in lower frequency (Figure 1), have also been detected as causative agents of conjunctivitis and blepharoconjunctivitis [53]. Finally, the identification of erythromycin- and penicillin-resistant strains marks the importance of detecting these resistance markers, as much of empirical antimicrobial therapy is based on the use of these antimicrobials [54,55]. Certainly, Gram-negative bacteria isolated from ocular anatomical sources are worthy of study because of their contribution to the dramatic increase in the emergence of multidrug-resistant strains. The presence of *P. luteola*, *Acinetobacter radioresistens*, and *Morganella morganii* mark the importance of their identification through bacteriological and susceptibility testing, as they have been reported as causative agents of postoperative endophthalmitis [56,57] and orbital abscess due to dacryocystitis [58]. These findings already highlight the important role of ocular commensal bacteria as potential disseminators of carbapenem-resistant genes. As shown in Figure 2B, b-lactam antibiotics continue to be the therapeutic options with the least antimicrobial activity, in contrast to other antibiotics, such as chloramphenicol, which is still used ophthalmologically for the treatment of ocular infections even though it is contraindicated due to its cytotoxicity [59]. 

Although antibiotics from the other antibiotic families have shown greater activity against Gram-negative bacteria (gentamicin, netilmicin, and amikacin), their therapeutic use in the treatment of ocular infections is limited. Even though the diagnosis of eye infections is primarily clinical, the support of diagnostic methods such as microbiological examinations, Gram staining or molecular methods is of great importance in certain situations where it is necessary to differentiate between bacterial cells or fungal structures, or in cases where the evolution of the disease can be rapid, such as in hyperacute conjunctivitis, ophthalmia neonatorum, keratitis, and endophthalmitis, in order to provide early and effective treatment [60]. It is also important to perform microbiological culture when there is a poor response to empirical treatment or if it is a chronic infection. While Gram staining has a low sensitivity in corneal scrapings, in early or small ulcers and more advanced ulcers, its result may have clinical value and may rule out colonising microbiota. Molecular methods are useful when keratitis due to *Acanthamoeba* or other bacteria can be mistaken for keratitis of viral origin, which delays treatment [61].

Currently, if *Chlamydia* infection is suspected, direct immunofluorescence tests, culture or PCR detection can be performed, facilitating the aetiological diagnosis [62]. Microbiological investigation of the aetiological agent of eye infections, together with antibiogram, should be mandatory for the selection of a specific antimicrobial therapy to prevent and/or stop the development of antibiotic resistance. Increasing resistance to these antibiotics currently represents a serious therapeutic problem and can lead to mild to severe visual impairment if the infectious process is not effectively treated.

## Figures and Tables

**Table 1:** Primers used in this study.

Primer	Molecular Target	Sequence (5′→3′)	Size (bp)	References
*mecA* plus	*mecA*	TGGCTATCGTGTCACAATCG	310	[24]
*mecA* minus		CTGGAACTTGTTGAGCAGAG		
16S plus	*16S rRNA*	AGGAGGTGATCCAACCGCA	370	
16S menos		AACTGGAAGAAGGTGGGGAT		
b	*ccrA2-B*	ATTGCCTTGATAATAGCCYTCT	937	[25]
a		TAAAGGCATCAATGCACAAACAC		
ccrCF	*ccrC*	CGTCTATTACAAGATGTTAAGGATAAT	518	
ccrCR		CCTTTATAGACTGGATTATTCAAAATAT		
1272F1	*IS1272*	GCCACTCATAACATATGGAA	415	
1272R1		CATCCGAGTGAAACCCAAA		
5RmecA	*mecA-IS431*	TATACCAAACCCGACAACTAC	359	
5R431		CGGCTACAGTGATAACATCC		
icaAF	*icaA*	ACACTTGCTGGCGCAGTCAA	188	[26]
icaAR		TCTGGAACCAACATCCAACA		
icaDF	*icaD*	ATGGTCAAGCCCAGACAGAG	900	
icaDR		AGTATTTTCAATGTTTAAAGCAA		

**Table 2:** Description of the signs/symptoms in patients with external ocular infections at Hospital Juárez de México.

Sign/Symptom	*n* (%)	C.I. 95%
Asymptomatic	6 (5.45)	(1.21–9.69)
Itchy	49 (44.5)	(35.21–53.79)
Tearing	7 (6.4)	(1.83–10.97)
Secretion	14 (12.7)	(6.49–18.92)
Burning	17 (15.5)	(8.74–22.26)
Decreased visual acuity	6 (5.5)	(1.24–9.76)
Foreign body feeling	29 (26.4)	(18.16–34.64)
Conjunctival hyperaemia	8 (7.3)	(2.44–12.16)
Pain	6 (5.5)	(1.21–9.69)
Others	6 (5.5)	(1.21–9.69)

**Table 3:** Description of the comorbidities in patients with external ocular infections from Hospital Juárez de México.

Comorbidities or Antecedent	*n* (%)	C.I. 95%
No comorbidities	20 (18.2)	(10.99–25.41)
*Diabetes mellitus* type 2	36 (32.7)	(23.93–41.47)
Cancer	11 (10.0)	(4.39–15.61)
Systemic arterial hypertension	34 (30.9)	(22.26–39.54)
Autoimmune diseases	17 (15.5)	(8.74–22.26)
Ocular surgery	29 (26.4)	(18.16–34.64)
Other ocular pathologies	41 (37.3)	(28.26–46.34)

**Table 4:** Distribution of *mecA* gene and mobile genetic elements *SCCmec* in *Staphylococcus* strains (methicillin resistant) isolated from external ocular infection cases from Hospital Juárez de México.

Specie of *Staphylococcus*	*mecA* *Gene*	*SCCmec* *n* (%)	
		III	IV
*Staphylococcus epidermidis* (*n* = 19)	+	12 (63.1)	7 (36.9)
*Staphylococcus haemolyticus* (*n* = 6)	+	5 (83.3)	1 (16.7)
*Staphylococcus aureus* (*n* = 3)	+	1 (33.3)	2 (66.6)
*Staphylococcus warneri* (*n* = 1)	+	1 (100)	0 (0)

## Data Availability

Not applicable.
